# Does Resistant Starch Formed by Cooling Pasta Decrease the Postprandial Glycemic Response in Type 1 Diabetes? A Randomized Single-Blind Crossover Study

**DOI:** 10.3390/nu18071152

**Published:** 2026-04-03

**Authors:** Anita Rogowicz-Frontczak, Sylwia Strozyk, Stanislaw Pilacinski, Anna Koperska, Joanna Le Thanh-Blicharz, Magdalena Tanska, Dorota Zozulinska-Ziolkiewicz

**Affiliations:** 1 Department of Internal Medicine and Diabetology, Poznan University of Medical Sciences, 60-834 Poznan, Poland; pilacins@ump.edu.pl (S.P.); dzozulinskaziolkiewicz@ump.edu.pl (D.Z.-Z.); 2 Institute of Health Sciences, Pomeranian University in Slupsk, 76-200 Slupsk, Poland; sylwia.strozyk@upsl.edu.pl (S.S.); magdalena.tanska@upsl.edu.pl (M.T.); 3 Department of Treatment of Obesity, Metabolic Disorders and Clinical Dietetics, Poznan University of Medical Sciences, 60-569 Poznan, Poland; anna.koperska@student.ump.edu.pl; 4 Department of Food Concentrates and Starch Products, Prof. Waclaw Dabrowski Institute of Agriculture and Food Biotechnology—State Research Institute, 02-532 Poznan, Poland; joanna.lethanh-blicharz@ibprs.pl

**Keywords:** type 1 diabetes, postprandial glycemia, resistant starch, retrogradation, continuous glucose monitoring

## Abstract

**Background:** Carbohydrate quality and culinary processing can meaningfully alter postprandial glycemia in people with type 1 diabetes (T1D). Cooling gelatinized starch promotes retrogradation and increases resistant starch (RS), potentially attenuating postprandial glucose excursions. **Objectives:** We investigated whether pasta cooled after cooking (24 h at 4 °C) and reheated before consumption improves postprandial glycemia in adults with T1D without increasing hypoglycemia risk under routine insulin pump bolus-calculator dosing. **Methods:** In this randomized, single-blind, crossover study, 32 adults with T1D treated with continuous subcutaneous insulin infusion (CSII) consumed two standardized pasta-based meals (50 g of available carbohydrate): freshly cooked pasta and cooled/reheated pasta. Participants administered rapid-acting insulin boluses calculated by their pump bolus calculator 10 min before the meal. Interstitial glucose was recorded for 180 min using flash glucose monitoring. **Results:** Compared with freshly cooked pasta, cooled/reheated pasta produced lower maximum glycemia (10.7 vs. 12.6 mmol/L, *p* = 0.0001), lower maximum glycemic rise (2.8 vs. 4.7 mmol/L, *p* < 0.0001), lower incremental area under the curve (iAUC; 211.9 vs. 524.8 mmol/L × 180 min, *p* < 0.0001), and a shorter time-to-peak (65 vs. 125 min, *p* = 0.014). Resistant starch content increased after cooling (12.88 ± 0.06 vs. 8.03 ± 0.08 g/100 g). The number of hypoglycemic episodes did not differ between conditions. **Conclusions:** Cooling and reheating pasta therefore increased RS and attenuated postprandial glycemia in adults with T1D without increasing early postprandial hypoglycemia in the studied setting.

## 1. Introduction

Type 1 diabetes (T1D) is an autoimmune disease characterized by destruction of pancreatic beta cells and lifelong dependence on exogenous insulin. Improving glycemic control while minimizing hypoglycemia is a central objective because sustained near-normoglycemia is associated with fewer chronic complications and better quality of life [1,2,3].

Postprandial glycemia is a key contributor to overall glycemic exposure and glycemic variability. Even with modern insulin analogs and insulin pump therapy, matching insulin action to the time course of glucose appearance after meals remains challenging, particularly with starchy foods [3,4]. Carbohydrate counting and pump bolus calculators are widely used to guide meal insulin dosing; however, two meals containing the same amount of carbohydrate can elicit different glycemic responses because of differences in food structure, processing, starch fractions (rapidly available vs. slowly available), and the effect of non-carbohydrate components (fat, protein, fiber) on gastric emptying and intestinal digestion [4,5,6,7].

Resistant starch (RS) is the fraction of dietary starch that escapes digestion in the small intestine and undergoes fermentation in the colon. Type 3 resistant starch (RS3) forms when gelatinized starch is cooled, a process that promotes retrogradation and increases crystalline, enzyme-resistant structures of amylose and amylopectin [8,9,10,11,12]. In people without diabetes, RS-enriched foods and household approaches such as cooling and reheating cooked starches are often associated with smaller postprandial glucose and insulin responses, although the magnitude of the effect depends on the food matrix, amylose content, cooling duration, and number of heating–cooling cycles [13,14,15,16].

Translating these approaches to T1D requires caution because endogenous insulin secretion cannot adapt to slower or reduced carbohydrate absorption. In our previous randomized crossover study of cooled rice in adults with T1D (FreeStyle Libre-based monitoring), cooling reduced postprandial glycemia but increased the frequency of hypoglycemia when insulin doses were not adjusted [17]. This implies that RS-based strategies may require food-specific insulin dosing to ensure safety.

Pasta is commonly consumed, is practical to standardize, and generally has a lower glycemic impact than many other refined starch sources because of its compact structure and slower starch hydrolysis [18,19]. Whether cooling and reheating pasta further improves postprandial glycemia in adults with T1D—and whether this alters early hypoglycemia risk under routine pump bolus-calculator dosing—has not been established.

Therefore, the primary aim of this study was to assess whether pasta cooled for 24 h at 4 °C and reheated before consumption reduces postprandial glycemia in adults with T1D. Secondary aims were to evaluate hypoglycemic episodes, organoleptic acceptability, and appetite-related responses.

## 2. Materials and Methods

### 2.1. Study Design and Ethical Approval

This clinical study was a randomized, single-blind, crossover trial. The Bioethics Committee of Poznan University of Medical Sciences approved the study (ethical approval no. 198/18; 1 February 2018). All participants provided written informed consent, and the study was conducted in accordance with the Declaration of Helsinki. The study was not prospectively registered in a public clinical trial registry.

### 2.2. Participants

Thirty-two adults with T1D were recruited from the Department of Internal Medicine and Diabetology, Poznan University of Medical Sciences (Poznan, Poland). Inclusion criteria were: (1) diagnosis of T1D; (2) age ≥ 18 years; (3) intensive insulin therapy using a personal insulin pump (CSII) with a bolus calculator function; (4) body mass index (BMI) < 30 kg/m^2^; (5) HbA1c < 9% (75 mmol/mol); and (6) provided written informed consent. Exclusion criteria were: (1) pregnancy; (2) diabetes types other than T1D; (3) eating disorders; (4) food allergy or intolerance to ingredients of the standardized meal; (5) history of celiac disease; (6) autonomic neuropathy, including suspected gastroparesis; and (7) insulin pump therapy duration < 3 months.

### 2.3. Baseline Assessments

Body composition was assessed using bioelectrical impedance analysis (TANITA BC-418 MA, Tanita Corporation, Tokyo, Japan).

Laboratory tests included HbA1c and lipid profile (total cholesterol, LDL cholesterol, HDL cholesterol, triglycerides) measured on a Cobas 6000 analyzer (Roche Diagnostics, Basel, Switzerland) using standard methods.

### 2.4. Randomization, Blinding and Study Visits

Participants consumed both test meals in randomized order. Randomization was performed using a computer-generated sequence that assigned participants in a 1:1 ratio to one of two meal-order sequences (fresh pasta followed by cooled/reheated pasta or cooled/reheated pasta followed by fresh pasta). The allocation sequence only determined the order of meal administration. Participants were blinded to the meal condition because the cooled pasta was reheated to match the serving temperature of the fresh pasta and was served with the same tomato sauce. The two test days were separated by at least 7 days and were scheduled at the same time of day for each participant.

### 2.5. Pre-Test Standardization

Meal tests were performed at 14:00 after a 5 h fasting interval (water allowed). Participants were instructed to avoid unusual vigorous physical activity beginning on the day before each test and to avoid exercise during the test. A test meal could be administered only if the participant had no hypoglycemia during the preceding 24 h and pre-meal glucose was 3.9–10.0 mmol/L. To minimize the influence of basal insulin infusion on the measured postprandial profile, each participant verified basal-rate stability between 13:00 and 17:00 prior to study initiation; if a non-optimal basal rate caused substantial glucose drift, basal settings were modified under medical supervision. On test days, basal rates were not changed.

### 2.6. Insulin Dosing

All participants used rapid-acting insulin analogs (insulin lispro or aspart) delivered via CSII. Ten minutes before each test meal, participants administered a bolus calculated using the pump bolus calculator (carbohydrate ratio, correction factor and insulin sensitivity factor). There was no active insulin remaining from a previous bolus at the time of test-meal administration.

### 2.7. Test Meals and Pasta Preparation

Each participant consumed two standardized pasta-based test meals on separate days in randomized order: (i) freshly cooked wheat pasta; (ii) wheat pasta cooled for 24 h at 4 °C and reheated immediately before consumption. Test meals consisted of 200 g of cooked wheat pasta served with 125 g of tomato sauce (no seasoning). Total available carbohydrate content was standardized to 50 g (45 g from pasta and 5 g from sauce) using food composition tables [20].

Pasta preparation was standardized: 100 g of dry pasta was boiled in 750 mL of water for 10 min using an induction cooker, drained, and portioned into 200 g servings based on cooked weight. For the cooled condition, the cooked pasta portion was refrigerated at 4 °C for 24 h and reheated by immersion in hot water for 3 min immediately before serving. To maintain blinding, reheated pasta was served at a similar temperature and with the same sauce as the fresh pasta.

### 2.8. Resistant Starch Measurement

Resistant starch content in fresh pasta and cooled/reheated pasta was determined using the AOAC 2002.02 method [21].

### 2.9. Continuous Glucose Monitoring and Outcomes

Interstitial glucose was recorded for 180 min using flash glucose monitoring (FreeStyle Libre 2, Abbott Diabetes Care Ltd., Witney, Oxfordshire, UK) [22].

The sensor was applied at least 48 h before the first test and was worn throughout the study period. Data were collected at 5 min intervals [23]. The primary endpoint was the 180 min incremental area under the interstitial glucose curve (iAUC). Secondary outcomes included maximum postprandial glucose, maximum glucose rise from baseline, time to peak (TTP), hypoglycemic episodes, organoleptic ratings, and appetite-related outcomes (hunger, satiety, and desire to eat).

### 2.10. Hypoglycemia Definition and Management

Hypoglycemia was defined as sensor glucose < 3.9 mmol/L and/or typical symptoms. Events were confirmed by capillary glucose measurement (Optium Xido meter, Abbott Diabetes Care, Alameda, CA, USA) when feasible. If hypoglycemia occurred, the test was paused, and participants ingested 15–20 g of fast-acting carbohydrates; glucose was rechecked after 15 min, and the time of the event was recorded.

### 2.11. Organoleptic Evaluation and Appetite Ratings

Participants rated taste (palatability), visual appeal, smell, and consistency using a 0–10 numerical scale after each meal. Hunger, satiety, and desire to eat were rated using a 0–10 visual analogue scale (VAS) before the meal and at 30, 60, 120, and 180 min postprandially.

### 2.12. Breakfast Control

Participants were instructed to consume the same breakfast on each test day. Breakfast composition (energy, carbohydrate, protein, fat, fiber) and breakfast insulin dose were recorded and analyzed using Dietician 2014 software with a Polish food database.

### 2.13. Statistical Analysis

Continuous variables were presented as mean ± SD for normally distributed data and as median (IQR) where the data were skewed.

The primary endpoint was iAUC over 180 min. Paired comparisons between fresh and cooled/reheated conditions used paired t-tests for normally distributed variables or Wilcoxon signed-rank tests for nonparametric data. Given the exploratory nature of the study, no formal correction for multiple comparisons was applied. Formal testing for period and carry-over effects was not performed. Two-sided *p* values < 0.05 were considered statistically significant. Data were analyzed using Statistica PL version 12.

## 3. Results

### 3.1. Participant Characteristics

Thirty-two participants completed both meal tests. Baseline characteristics are presented in Table 1. None of the patients were smokers, had food allergies or intolerances, or were diagnosed with chronic complications of diabetes. The results were presented as n (%), mean ± SD or median (IQR).

### 3.2. Test Meal Composition and Resistant Starch

Nutrient composition of the wheat pasta used for the test meal is shown in Table 2. Resistant starch content increased after cooling and reheating (Table 3).

### 3.3. Postprandial Glucose Response

**Figure 1:**
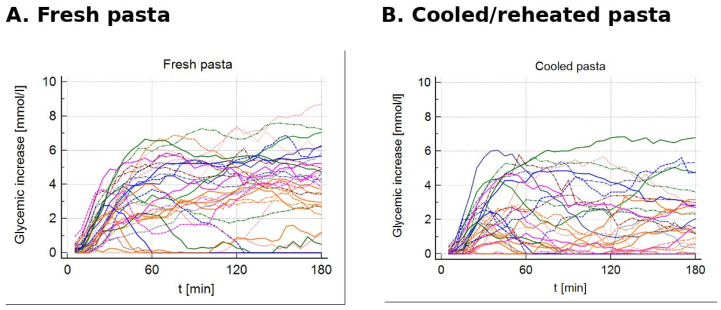
Individual interstitial glycemic increase curves relative to baseline over 180 min after (**A**) fresh pasta and (**B**) cooled/reheated pasta test meals. Each colored line represents one individual participant.

Compared with freshly cooked pasta, cooled/reheated pasta resulted in significantly lower maximum glycemia, lower maximum glycemic rise, lower iAUC, and shorter time to peak. A graphical presentation of the individual glycemic increase curves during the 180 min observation period is shown in Figure 1; summary statistics are presented in Table 4.

### 3.4. Insulin Doses and Breakfast Intake

Insulin doses did not differ between test conditions. The meal bolus dose was 5.3 (5.0–6.0) units for fresh pasta and 5.4 (4.9–6.0) units for cooled/reheated pasta (*p* = 0.83). Basal insulin delivery on the study days did not differ between conditions [fresh pasta: 15.8 (13.2–19.9) units vs. cooled/reheated pasta: 15.8 (13.2–19.9) units; *p* = 0.42]. Breakfast composition and breakfast bolus dose were comparable on both test days (Table 5).

### 3.5. Hypoglycemic Episodes

The number of postprandial hypoglycemic episodes within 180 min did not differ between conditions. One participant (3.1%) experienced hypoglycemia 170 min after the fresh pasta meal. Two participants (6.3%) experienced hypoglycemia after the cooled/reheated pasta meal at 90 and 150 min.

### 3.6. Organoleptic Evaluation

Organoleptic ratings were similar for fresh and cooled/reheated pasta (Table 6).

### 3.7. Appetite Ratings

Hunger ratings were slightly higher and satiety ratings slightly lower at 120 min after cooled/reheated pasta compared with fresh pasta; no significant differences were observed at the other time points. Desire-to-eat ratings did not differ at any time point. These appetite-related findings should be interpreted as exploratory (Table 7, Table 8 and Table 9).

## 4. Discussion

In this randomized, single-blind crossover trial in adults with T1D treated with CSII, cooling pasta for 24 h at 4 °C and reheating it before consumption was associated with a more favorable early postprandial glycemic profile than freshly cooked pasta. Cooled/reheated pasta produced a lower glucose peak, a smaller maximum glycemic rise, a substantially lower iAUC, and an earlier time to peak. Importantly, these differences were observed without a clear increase in early postprandial hypoglycemic episodes under standard pump bolus-calculator dosing. Together, these findings suggest that a simple household preparation strategy may meaningfully modify the glycemic impact of a commonly consumed food in adults with T1D.

### 4.1. Mechanistic Interpretation

The most plausible explanation for the observed differences is the increase in resistant starch after cooling. Cooking in excess water gelatinizes starch and increases enzymatic accessibility. Cooling promotes retrogradation, during which, amylose molecules and long branch chains of amylopectin form ordered structures (double helices and crystallites) that resist alpha-amylase hydrolysis [8,9,10,11,12]. As a result, a portion of starch behaves like dietary fiber in the small intestine, lowering the rapidly available carbohydrate fraction and slowing glucose appearance in the circulation. In our study, resistant starch content increased by approximately 60% in cooled/reheated pasta, supporting a mechanistic link between RS formation and the observed reduction in glucose excursions.

### 4.2. Comparison with Evidence from Non-Diabetic Populations

In people without diabetes, multiple lines of evidence indicate that the preparation method modifies glycemic responses to starchy foods even when the carbohydrate content remains unchanged. Hodges et al. reported that serving a carbohydrate-rich meal containing pasta after chilling or reheating altered the postprandial glucose profile compared with serving the same meal hot, supporting the concept that household processing changes starch digestibility [14]. Robertson et al. observed cumulative glycemic benefits of chilling and reheating a pasta-based meal in a crossover pilot study [15]. Analogous findings have been reported for rice and potatoes: cooling and/or reheating can increase resistant starch and lower postprandial glycemia in controlled studies, although the magnitude varies by food type and experimental design [24,25,26,27]. These studies collectively suggest that retrogradation-based strategies are not unique to a single starch source but that the food matrix strongly shapes the metabolic outcome.

### 4.3. Food-Matrix Specificity and Implications for Insulin Dosing in T1D

In T1D, the clinical value of resistant-starch strategies depends not only on reducing postprandial hyperglycemia but also on maintaining safety, particularly with respect to hypoglycemia. In our prior crossover study of cooled rice in adults with T1D, cooling reduced postprandial glycemia but significantly increased hypoglycemic episodes during the 180 min observation period when insulin dosing was not adjusted [17]. This likely reflects a mismatch between exogenous insulin action—dosed and timed for rapidly digestible starch—and a reduced or delayed carbohydrate appearance profile after cooling. In contrast, in the present pasta study, the same general approach improved glycemia without increasing early hypoglycemia under unchanged insulin doses.

Several factors may explain these different safety profiles. First, pasta typically induces a slower glycemic rise than many rice preparations because of its compact structure and slower starch hydrolysis [18,19]. A slower carbohydrate appearance profile may align more closely with the pharmacokinetics of rapid-acting insulin delivered 10 min before the meal. Second, the absolute and relative increase in resistant starch may differ between rice and pasta depending on amylose content and the cooling protocol; studies in non-diabetic populations confirm that both variety and preparation method influence resistant starch formation and glycemic response [24,25,27,28]. Third, the meal matrix in this protocol (pasta with tomato sauce) may have modestly slowed absorption through viscosity and acidity, thereby buffering against rapid excursions and insulin–glucose mismatch.

From a practical standpoint, our findings suggest that cooled/reheated pasta may represent a promising dietary strategy to attenuate postprandial glucose excursions in adults with T1D without automatically reducing the meal bolus, at least for the tested meal size and carbohydrate content. However, individual variability in insulin sensitivity, gastric emptying, and bolus timing is substantial. Patients who adopt this approach should review postprandial glucose patterns and, if necessary, tailor bolus type (standard vs. extended/dual wave) or dose, particularly when combining the pasta with additional fat or protein. Because carbohydrate appearance can also be influenced by the composition of the preceding meal and by within-day factors, consistent monitoring remains essential [6,29].

### 4.4. Standardization and Factors Influencing Postprandial Responses

Postprandial glycemia in T1D is influenced by basal insulin infusion, residual insulin on board, pre-meal glucose, and the composition of prior meals. We standardized meal timing (14:00), fasting interval (5 h), available carbohydrate (50 g), and instructed participants to avoid unusual physical activity. Basal-rate stability in the test window was verified prior to study initiation, and basal rates were not changed on test days. To reduce the potential second-meal effect and day-to-day variability, participants consumed the same breakfast on each test day, and breakfast macronutrients were recorded; no clinically relevant differences were observed between test days. These controls increase confidence that the observed differences arose primarily from the pasta preparation method and associated changes in resistant starch content.

### 4.5. Acceptability and Appetite-Related Outcomes

Implementation of preparation-based strategies depends on acceptability. Organoleptic ratings were high and did not differ between fresh and cooled/reheated pasta, indicating that the cooling protocol did not compromise palatability, appearance, smell, or texture. Appetite-related ratings were largely similar; the differences observed in hunger and satiety at 120 min may reflect variability rather than a robust physiological effect. In experimental studies, appetite effects of resistant starch have been inconsistent and may depend on RS type, dose, and fermentation-related mechanisms rather than on the modest RS increase achieved by a single cooling cycle [16,29]. Accordingly, the appetite-related findings of the present study should be regarded as exploratory. Longer-term studies capturing subsequent meal intake and longer glucose monitoring windows could clarify whether cooled/reheated pasta meaningfully affects satiety and energy intake in T1D.

### 4.6. Limitations

Several limitations should be considered when interpreting these findings. First, this was an exploratory, investigator-initiated single-center crossover study, and no formal a priori sample size calculation was performed. The final sample size was determined pragmatically based on recruitment feasibility and the number of eligible participants available during the study period. The crossover design increased analytical efficiency by allowing each participant to serve as his or her own control, thereby reducing inter-individual variability.

Second, interstitial glucose was assessed using flash glucose monitoring. Because interstitial glucose lags blood glucose during periods of rapid glycemic change, absolute peak glucose values and time-to-peak may have been affected.

However, the same monitoring method was used under both conditions in a within-subject crossover design, supporting the validity of the relative comparison between meals. Third, participants were able to view sensor readings during the study period, which may have partially compromised blinding and influenced expectations or symptom awareness. Fourth, formal testing for period and carry-over effects was not performed, although the acute meal-based intervention and the washout interval of at least 7 days make biologically meaningful carry-over unlikely. Fifth, we tested only one pasta type and one cooling protocol; resistant starch formation varies according to formulation, amylose content, cooling duration, and the number of heating–cooling cycles. Sixth, the observation period was limited to 180 min and did not capture later postprandial excursions, delayed hypoglycemia, time-in-range, time-above-range, or short-term glycemic variability. Seventh, the study was performed under standardized experimental meal conditions and therefore may not fully reflect real-world eating patterns. Finally, the study was not prospectively registered in a public clinical trial registry. Taken together, these limitations indicate that the findings should be considered exploratory and hypothesis-generating. Future studies should evaluate other pasta formulations, quantify broader CGM-derived metrics, and examine insulin-delivery strategies such as extended or dual-wave boluses to better match insulin action to carbohydrate appearance in resistant-starch-enriched meals. In addition, no formal correction for multiple comparisons was applied; these issues should therefore be considered methodological limitations of this exploratory study.

## 5. Conclusions

Cooling and reheating pasta increased resistant starch content and significantly reduced early postprandial glucose excursions in adults with T1D treated with CSII. Unlike cooled rice—where reduced postprandial glycemia was accompanied by increased hypoglycemia under unchanged insulin dosing—cooled/reheated pasta did not increase early postprandial hypoglycemia in the tested setting. Cooled/reheated pasta may therefore represent a promising practical strategy to attenuate postprandial glycemia in T1D; however, these findings should be regarded as exploratory and require confirmation in larger, adequately powered studies with longer monitoring periods. Accordingly, these results should be interpreted with caution until they are confirmed in larger, adequately powered studies with longer monitoring periods and broader glycemic endpoints.

## Figures and Tables

**Table 1:** Baseline characteristics of participants.

Variable	Result (n = 32)
Sex (female/male), n (%)	16/16 (50/50)
Age (years), median (IQR)	24 (22.5–29.5)
Duration of diabetes (years), mean ± SD	11.5 ± 5.7
Duration of CSII use (years), mean ± SD	6.0 ± 3.7
BMI (kg/m^2^), mean ± SD	22.7 ± 2.3
Total body fat (%), mean ± SD	20.1 ± 7.7
HbA1c (%), mean ± SD	6.9 ± 0.6
HbA1c (mmol/mol), mean ± SD	52 ± 7
Total cholesterol (mmol/L), mean ± SD	4.23 ± 0.80
LDL cholesterol (mmol/L), median (IQR)	1.79 (1.42–2.40)
HDL cholesterol (mmol/L), mean ± SD	1.93 ± 0.39
Triglycerides (mmol/L), median (IQR)	0.64 (0.53–0.89)

**Table 2:** Nutrient composition of wheat pasta used for the test meal (per 100 g of cooked pasta) [20].

Component (Per 100 g of Cooked Pasta)	Value
Energy (kcal)	111
Protein (g)	3.5
Fat (g)	0.5
Carbohydrate (g)	22.8
Starch (g)	22.3
Dietary fiber (g)	0.8

**Table 3:** Resistant starch content in fresh and cooled/reheated pasta (AOAC 2002.02) [21]. Values are mean ± SD.

	Fresh Pasta	Cooled/Reheated Pasta (24 h at 4 °C)
Resistant starch (g/100 g)	8.03 ± 0.08	12.88 ± 0.06

**Table 4:** Postprandial glucose metrics after test meals. Values are median (IQR).

Outcome	Fresh Pasta	Cooled/Reheated Pasta	*p* Value
Maximum glycemia (mmol/L)	12.6 (12.1–13.6)	10.7 (9.3–12.7)	0.0001
Maximum glycemic rise, 180 min (mmol/L)	4.7 (3.6–6.2)	2.8 (1.8–4.9)	0.0001
iAUC, 180 min (mmol/L × 180 min)	524.8 (313.9–760.8)	211.9 (81.2–512.1)	0.0001
Time to peak (min)	125 (55–170)	65 (38–140)	0.014
Maximum glycemic rise, 120 min (mmol/L)	3.9 (3.1–5.4)	2.6 (1.6–4.6)	0.0001
iAUC, 120 min (mmol/L × 120 min)	289.1 (217.2–456.6)	113.6 (56.0–333.1)	0.0001

**Table 5:** Breakfast intake prior to test meals. Values are median (IQR).

Variable	Fresh Pasta Day	Cooled/Reheated Pasta Day	*p* Value
Breakfast bolus insulin (units)	6.0 (3.8–7.7)	5.5 (3.9–7.7)	0.66
Energy (kcal)	403.6 (312.6–471.9)	402.5 (312.6–466.5)	0.66
Protein (g)	17.3 (11.8–18.9)	17.8 (11.8–18.9)	0.66
Fat (g)	15.9 (12.3–19.5)	15.5 (12.3–19.5)	0.18
Carbohydrate (g)	48.1 (40.3–68.3)	47.8 (42.9–68.3)	0.66
Dietary fiber (g)	7.8 (4.5–8.3)	7.8 (4.5–8.3)	0.66

**Table 6:** Organoleptic assessment of test meals (0–10 scale). Values are median (IQR).

Attribute	Fresh Pasta	Cooled/Reheated Pasta	*p* Value
Palatability	7 (6–8)	8 (6–9)	0.14
Visual appeal	7 (5–9)	7 (6–8)	0.99
Smell	8 (6–9)	8 (7–9)	0.73
Consistency	7 (6–8)	7 (6–8)	0.40

**Table 7:** Hunger ratings (VAS 0–10). Values are median (IQR).

Time Point	Hunger: Fresh	Hunger: Cooled/Reheated	*p* Value
Before meal	7 (6–9)	7 (7–9)	0.28
30 min	3 (2–5)	4 (2–6)	0.41
60 min	5 (3–7)	6 (3–7)	0.19
120 min	5 (2–7)	6 (3–7)	0.021
180 min	7 (5–8)	7 (4–8)	0.78

**Table 8:** Satiety ratings (VAS 0–10). Values are median (IQR).

Time Point	Satiety: Fresh	Satiety: Cooled/Reheated	*p* Value
Before meal	3 (1–4)	3 (1–4)	0.82
30 min	6 (5–8)	7 (5–8)	0.63
60 min	5 (4–7)	6 (4–8)	0.87
120 min	5 (4–8)	4 (3–7)	0.035
180 min	3 (2–8)	3 (2–5)	0.30

**Table 9:** Desire-to-eat ratings (VAS 0–10). Values are median (IQR).

Time Point	Fresh Pasta	Cooled/Reheated Pasta	*p* Value
Before meal	8 (6–9)	7 (7–9)	0.80
30 min	5 (2–7)	6 (2–7)	0.99
60 min	6 (4–8)	6 (3–7)	0.09
120 min	7 (3–8)	6 (3–8)	0.80
180 min	8 (4–8)	7 (4–9)	0.27

## Data Availability

De-identified individual participant data are not publicly available because the dataset includes individual-level clinical and glucose-monitoring data from a relatively small cohort, which may increase the risk of participant re-identification. Data may be made available by the corresponding author upon reasonable request, subject to institutional, ethical, and privacy restrictions.

## Abbreviations

AUC, area under the curve; BMI, body mass index; CSII, continuous subcutaneous insulin infusion; CGM, continuous glucose monitoring; iAUC, incremental area under the curve; IQR, interquartile range; RS, resistant starch; RS3, resistant starch type 3; T1D, type 1 diabetes; TTP, time to peak; VAS, visual analogue scale.
